# Increasing incidence of normal pressure hydrocephalus in Germany: an analysis of the Federal Statistical Office Database from 2005 to 2022

**DOI:** 10.1038/s41598-024-79569-8

**Published:** 2024-12-23

**Authors:** Santhosh G. Thavarajasingam, Ahmed Salih, Srikar R. Namireddy, Florian Ringel, Andreas Kramer

**Affiliations:** 1 Department of Neurosurgery, University Medical Center Mainz, Langenbeckstraße 1, Mainz, Germany; 2 Imperial Brain & Spine Initiative, Imperial College London, London, UK; 3 Faculty of Medicine, Imperial College London, London, UK

**Keywords:** Epidemiology, Medical research, Outcomes research, Neurological disorders, Hydrocephalus

## Abstract

Normal pressure hydrocephalus (NPH) is a reversible cause of gait disturbances and dementia in the elderly, posing diagnostic and therapeutic challenges. In Germany, the epidemiology and surgical management of NPH are not well understood. This study aimed to characterise epidemiological trends and evaluate surgical management strategies for NPH in Germany. A retrospective nationwide population-based study of NPH cases in Germany from 2005 to 2022 was conducted using data from the German Federal Statistical Office. Parameters assessed included incidence trends, demographic characteristics, and surgical interventions. A total of 118,526 NPH diagnoses were recorded, with 29,662 surgical interventions. The population-adjusted incidence of NPH increased by 48%, from 5.4 to 8.0 cases per 100,000 individuals (*p* < 0.001), peaking in 2018. The largest increases were seen in the “80–89” age group, followed by the “70–79” and “90+” age groups. Surgical interventions increased by 8.4% (*p* < 0.001), with ventriculoperitoneal shunt being the predominant procedure. The study highlights a 48% rise in NPH incidence in Germany from 2005 to 2022, particularly affecting the elderly. There was also an increase in surgical interventions, underscoring the need for prioritising NPH in national healthcare research agendas.

## Introduction

Normal pressure hydrocephalus (NPH) is characterized by gait disturbances, urinary incontinence, and cognitive impairment, accompanied by ventriculomegaly but normal cerebrospinal fluid (CSF) pressure^1^. It is classified into idiopathic, predominantly affecting the elderly with unknown aetiology, and secondary NPH, associated with subarachnoid haemorrhage, meningitis, head trauma, or stroke, affecting various age groups^2^. NPH is notable for being a reversible form of dementia through CSF diversion surgery^3^. Untreated, it leads to progressive motor and cognitive decline, imposing significant clinical and socioeconomic burdens^4^. Hence timely recognition and management of this condition are pivotal^5^.

Advancements in diagnostic methodologies and an enriched understanding of NPH have notably decreased the incidence of undiagnosed cases. This evolution is critical for a precise evaluation of NPH’s actual burden, historically prone to underestimation^6^. Emerging epidemiological data, reflecting the advancements in diagnostics, reveal an increase in identified cases from a prevalence of 0.5% in individuals aged 67 and above in early studies, to up to 2.1% in those over 70 in recent analyses^7^. This trend underscores the urgency for contemporary epidemiological studies to accurately determine NPH’s impact. The 1995 Starnberg study in Bavaria, Germany, which employed door-to-door surveys, questionnaires, and neurological assessments, highlighted a higher incidence than contemporarily recognised^8^. With the refinement of diagnostic criteria and enhanced access to diagnostic facilities^9^, the potential for misclassification with other neurodegenerative conditions has been minimized, necessitating a re-evaluation of NPH’s epidemiological landscape in Germany to reflect its current prevalence and health implications accurately.

Previous studies, such as the one conducted by Lemcke et al. in 2016, provided preliminary insights into the prevalence of NPH using data from a subset of the population (> 10% of the overall population) covered by a specific insurance provider^10^. Their approach has several limitations, notably the absence of age-specific incidence rates, a limited view on the spectrum of surgical interventions for NPH, and lack of generalisability. Our study aims to characterise the epidemiology of NPH in Germany more reliably. Our study utilizes a nationally representative federal database, which encompasses exhaustive details of diagnoses and surgical interventions across German hospitals from 2005 to 2022.

## Methods

The hospital-referred reports from the Federal Office of Statistics (Statistisches Bundesamt/Destatis) database between 2005 and 2022 were analysed to characterise the incidence of Normal Pressure Hydrocephalus in Germany. The database contains details of all diagnoses and surgical interventions across German hospitals. The database offers big data which can be mined and analysed for trends and patterns in relevant quantitative and qualitative healthcare parameters, such as the epidemiological evolution of specific diseases. The database is openly available to the public and characterises hospital admission activity stratified by diagnostic (ICD-10) code. Furthermore, the available information includes treatment measures classified according to the official operation and procedure code (OPS). The OPS codes corresponding to specific ICD codes can be obtained from the linked database upon request.

The extracted codes include the number of diagnoses of Normal Pressure Hydrocephalus (total and age-stratified), surgical operations, and procedures clustered per year between 2005 and 2022.

The singular ICD-10 code G91.2 identified all cases of Normal-Pressure Hydrocephalus and encompassed three subcodes: G91.20 Idiopathic normal-pressure hydrocephalus, G91.21 Secondary normal-pressure hydrocephalus and G91.29 Normal-pressure hydrocephalus, unspecified. For the scope of our analysis, we scrutinised cases where the coded OPS codes, specifically 5-220 (“Incision on the cerebrospinal fluid system”), 5-023 (“Creating a CSF shunt”) and 5-024 (“Revision and removal of CSF drains”) that were directly associated with the ICD-10 diagnoses. This ensures that we observe surgical interventions explicitly linked to the Normal Pressure Hydrocephalus diagnosis. The incidence was then calculated using absolute mid-year population estimates and the relative distribution of age groups, sourced from Destatis.

### Statistical analysis

Descriptive statistics were used to summarize the data on diagnoses, the type of discharges, and mortality rates (Tables 1 and 2) for normal pressure hydrocephalus between 2005 and 2021, overall and per age group. To calculate the population-adjusted incidence, the Destatis data variables for each year were divided by the German population mid-year estimate of the respective year. Furthermore, to accurately understand the incidence of the disease amidst the epidemiological transition caused by demographic changes, the incidence was calculated for each age group separately by considering the annual proportional share of each age group in the total population. A trend analysis was performed to determine if there was a significant increase or decrease in the incidence over time. To examine the age distribution of patients with NPH, the mean age and IQR were calculated. To investigate further, a quantitative analysis over time was performed for the different surgical interventions associated with NPH were analysed, as well as the types of CSF shunt, and other types of surgical CSF diversion. To analyse the number of diagnoses, pairwise comparisons by age were made using t-tests with pooled standard deviation, adjusting the p-values using the Bonferroni method. The impact of age and year on NPH incidence was assessed using a two-way ANOVA, followed by Tukey’s test for post-hoc pairwise comparisons. Univariate linear regressions were carried out for each age group for diagnoses over time statistical analyses, and graph syntheses were conducted using the R software (version 4.0.4). A p-value of less than 0.05 was considered statistically significant (*p* < 0.05).

**Table 1:** Data extracted from the German Federal Statistical Office database for normal pressure hydrocephalus diagnoses (ICD-10: G19.2) between 2005 and 2022.

Year	Total number of diagnoses	Age group in years								
		0–19	20–29	30–39	40–49	50–59	60–69	70–79	80–89	90+
2005	4552	74	25	24	61	145	920	2286	976	41
2006	4457	72	9	26	40	152	852	2288	979	39
2007	5098	49	14	29	51	149	921	2596	1253	36
2008	5291	52	21	19	53	171	971	2644	1326	34
2009	5890	37	14	21	54	174	990	2976	1593	31
2010	5961	29	10	25	69	191	945	3046	1580	66
2011	6210	35	8	19	49	177	899	3338	1622	63
2012	6607	44	16	16	50	191	945	3562	1713	70
2013	6819	27	23	20	57	200	1040	3623	1761	68
2014	7473	29	21	20	44	213	1019	4112	1937	78
2015	7724	33	17	16	47	196	1011	4233	2087	84
2016	7942	25	15	23	47	201	1020	4295	2229	87
2017	8057	13	13	27	35	205	1039	4225	2397	103
2018	8230	23	14	22	44	205	1037	4218	2566	101
2019	8067	20	18	21	41	214	1060	3964	2629	100
2020	6653	17	6	18	35	197	900	3162	2242	76
2021	6751	13	13	19	24	190	890	3209	2302	91
2022	6744	14	14	22	35	152	949	3026	2465	67

A table showing the data extracted from the German Federal Statistical Office database for Normal Pressure Hydrocephalus diagnoses (ICD-10: G19.2) between 2005 and 2022. The following variables were extracted: Year, Age, number of diagnoses for normal pressure hydrocephalus.

**Table 2:** Data extracted from the German Federal Statistical Office database for normal pressure Hydrocephalus surgical interventions (ICD-10: G19.2) between 2005 and 2022.

Year	Total number of surgical interventions	CSF shunt					Revision and removal of CSF drains	Intracranial measuring probe	Incision on the cerebrospinal fluid system					Non-specified Surgical interventions
		Total	Ventriculoatrial	Ventriculoperitoneal	Telemetric shunt monitor	Other shunt			Total	CSF external drainage	Ventricular reservoir insertion	ETV	Other incisions	
2005	1346	904	55	833	0	16	178	127	124	78	23	17	6	13
2006	1279	829	37	778	0	14	188	158	82	49	17	16	0	22
2007	1374	976	38	925	0	13	164	112	108	59	27	17	5	14
2008	1440	974	16	953	0	5	212	99	133	66	44	20	3	22
2009	1670	1159	21	1131	0	7	202	183	106	37	40	26	3	20
2010	1496	1058	16	1025	3	14	194	99	126	29	71	26	0	19
2011	1651	1126	20	1087	5	14	218	148	132	41	71	20	0	27
2012	1736	1252	19	1218	6	9	192	147	124	33	68	23	0	21
2013	1817	1312	9	1292	0	11	235	128	113	39	63	11	0	29
2014	1874	1365	13	1314	21	17	257	102	134	45	63	20	6	16
2015	1830	1378	17	1319	27	15	230	75	122	34	59	16	13	25
2016	1856	1439	17	1401	17	4	248	51	99	23	54	13	9	19
2017	1943	1500	13	1452	21	14	269	41	110	24	70	10	6	23
2018	1835	1434	18	1389	10	17	216	53	114	16	76	14	8	18
2019	1901	1485	16	1452	14	3	199	71	129	20	74	17	18	17
2020	1519	1174	22	1148	4	0	179	35	116	22	70	11	13	15
2021	1636	1281	17	1247	13	4	181	18	135	29	82	15	9	21
2022	1459	1194	15	1171	8	0	145	10	102	14	77	6	5	8

A table showing the data extracted from the German Federal Statistical Office database for Normal Pressure Hydrocephalus surgical intervention (ICD-10: G19.2) between 2005 and 2022. The following variables were extracted: Year, Number of CSF shunt, Revision and removal of CSF drains, Intracranial measuring probe and Incision on the cerebrospinal fluid system.

*ETV* endoscopic third-ventriculostomy.

## Results

In total, the data analysed between 2005 and 2022 revealed 118,526 diagnoses of normal pressure hydrocephalus (ICD-10: G91.2) with 29,662 surgical interventions performed, including 21,840 CSF shunts performed, 3707 revisions and removals of CSF drains, 658 CSF external drainage systems implanted, and 298 endoscopic third ventriculostomies performed (Table 1).

### Total diagnoses and population-adjusted incidence

**Fig. 1:**
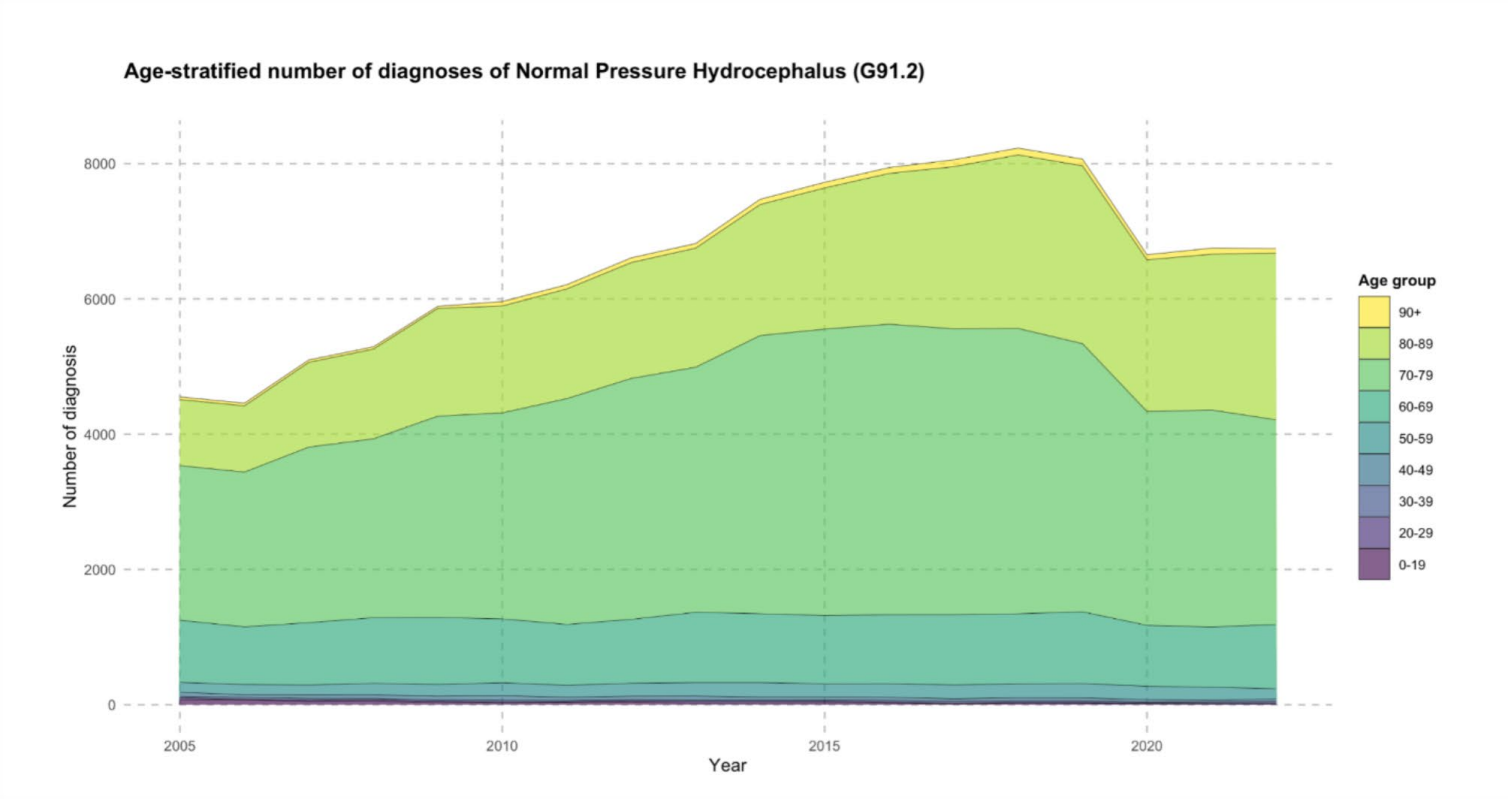
This area chart visually represents the number of normal pressure hydrocephalus diagnoses (categorized by ICD-10 code: G91.2) in Germany from 2005 to 2022. The distinct color layers correspond to different age groups, illustrating the distribution of these diagnoses across age groups. The vertical axis represents the number of total diagnoses from the general German population, while the horizontal axis spans the years under observation.

**Fig. 2:**
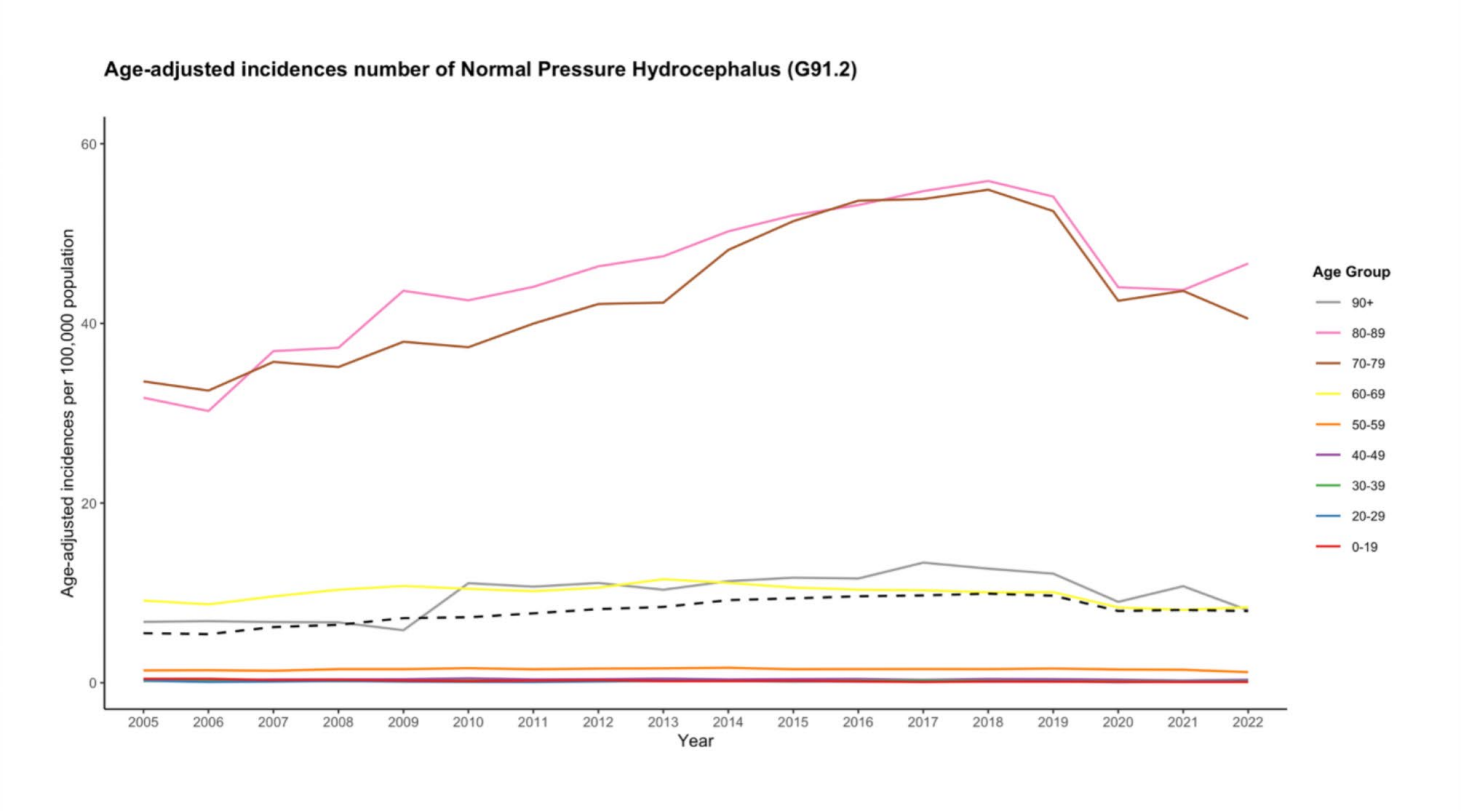
The presented line plots elucidate the age-adjusted incidence rates of normal pressure hydrocephalus diagnoses (categorized by ICD-10 code: G91.2) in Germany spanning the years 2005 to 2022, distinctly represented for various age groups. The black dashed line in both panels symbolizes the cumulative incidence rate across all age groups, offering a consolidated view of the disease’s prevalence each year.

Overall diagnoses for normal pressure hydrocephalus increased by 48.2%, from 4552 in 2005 to 6,744 in 2022 (Fig. 1). The peak was in the year 2018, with 8230 diagnoses. The population-adjusted incidence of normal pressure hydrocephalus for all age groups combined from 2005 to 2022 averaged 8.0 diagnoses per 100,000 population. The incidence increased by 44.8%, from 5.5 in 2005 to 8.0 in 2022 (Fig. 2) with the peak in 2018 at 9.9 diagnoses per 100,000 population (Table 1).

### Age group-stratified number of diagnoses

The 70–79 age group had the most diagnoses with 60,803 between 2005 and 2022 (51.3% of the total). This was followed by the 80–89 age group (33,657 diagnoses, 28.4%) and the 60–69 age group (17,408 diagnoses, 14.7%). The highest single-year diagnosis for normal pressure hydrocephalus in 2018 was for the 70–79 age group with 4,218 and the 80–89 age group with 2,566 diagnoses (Fig. 1).

### Age group-adjusted incidence

To characterize the rise in incidence against the epidemiological transition due to demographic change, we assessed the incidences for all age groups, accounting for their annual proportional share of the population. The top three age groups with significant relative changes from 2005 to 2022 were identified. The “80–89” age group experienced the most dramatic increase, with an incidence rate that went from 31.7 in 2005 to 46.7 per 100,000 in 2022 (47% relative change). This was followed by the “70–79” and “90+” age groups, with incidence rates of 40.5 and 8.1 in 2022, respectively (Fig. 2). In our investigation, three age groups demonstrated a rise in normal pressure hydrocephalus incidence from 2005 to 2022 that surpassed predictions based solely on demographic growth: 70–79, 80–89 and 90+. Conversely, the other age groups demonstrated a decline in incidences including the: 0–19, 20–29, 30–39, 40–49, 50–59 and 60–69 (Table 1). These results suggest that the rise in normal pressure hydrocephalus diagnoses is not solely attributable to population growth. A two-way ANOVA results revealed statistically significant effects of both age (F = 572.608, p < 0.001) and year (F = 3.619, p < 0.001) on the incidence. The comparisons within age groups indicated significant differences between various age groups (p < 0.001) (Table 3), while comparisons among years showed significant differences between multiple years (p < 0.001). The latter findings were confirmed by univariate analyses for age groups: 0–19, 40–49, 50–59, 70–79, 80–89 and 90 + with year as an explanatory variable (p < 0.05) (Table 4). These findings suggest that both age and year are important factors influencing the incidence of normal pressure hydrocephalus.

**Table 3:** Pairwise comparisons using t-tests with pooled SD for number of diagnoses of normal pressure Hydrocephalus surgical intervention (ICD-10: G19.2, 2005 to 2022) between different age groups.

Pairwise comparisons using t tests with pooled SD									
Data: Diagnoses–age									
	0–19	20–29	30–39	40–49	50–59	60–69	70–79	80–89	90+
Age 20–29	1	–	–	–	–	–	–	–	–
Age 30–39	1	1	–	–	–	–	–	–	–
Age 40–49	1	1	1	–	–	–	–	–	–
Age 50–59	1	1	1	1	–	–	–	–	–
Age 60–69	6.20E−07	3.40E−07	4.20E−07	9.40E−07	6.40E−05	–	–	–	–
Age 70–79	< 2e−16	< 2e−16	< 2e−16	< 2e−16	< 2e−16	2.00E−16	–	–	–
Age 80–89	< 2e−16	< 2e−16	< 2e−16	< 2e−16	< 2e−16	1.70E−06	3.70E−16	–	–
Age 90+	1	1	1	1	1	1.90E−06	< 2e−16	< 2e−16	–
Total	< 2e−16	< 2e−16	< 2e−16	< 2e−16	< 2e−16	< 2e−16	< 2e−16	< 2e−16	< 2e−16

P value adjustment method: Bonferroni.

**Table 4:** Univariate linear regression analysis of the relationship between the number of diagnoses of normal pressure Hydrocephalus surgical intervention (ICD-10: G19.2, 2005 to 2022) and several age factors.

Univariate linear regression			
lm (formula = Diagnoses–year)			
	Estimate	P value	Multiple R-squared co-efficient
Age 0–19	3.0877	5.42e−07***	0.8006
Age 20–29	− 0.2136	0.378	0.04881
Age 30–39	− 0.2239	0.183	0.1082
Age 40–49	− 1.4262	0.000952***	0.5048
Age 50–59	2.3849	0.0152*	0.316
Age 60–69	3.748	0.194	0.1032
Age 70–79	77.39	0.00804**	0.3241
Age 80–89	9.41E+01	13e−09***	0.9072
Age 90+	3.6419	4.61e−05***	0.6561
Total	176.29	0.000129***	0.6103

This table shows the results of linear regression analyses assessing the relationship between the number of diagnoses of Normal Pressure Hydrocephalus surgical intervention (ICD-10: G19.2, 2005 to 2022) and year, stratified by age groups. The table reports the regression coefficient estimates, p-values and Multiple R-squared co-efficient for each age group. The table includes ten rows, each representing a different age group, ranging from 0–19 to 90 and above, and a total row for all age groups combined.

Significance codes: 0 = ‘***’, 0.001 = ‘**’, 0.01 = ‘*’ 0.05 = ‘.’

### Total surgical interventions

**Fig. 3:**
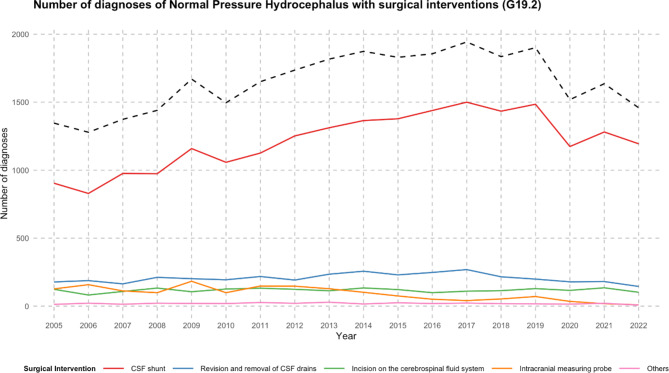
The presented line plots elucidate the number of surgical interventions for patients with normal pressure hydrocephalus (categorized by ICD-10 code: G91.2) in Germany between 2005 and 2022. The operations are classified into seven specific surgical categories: CSF shunt (OPS-2023: 5-023), incision on the cerebrospinal fluid system (OPS-2023: 5-220), revision and removal of CSF drains (OPS-2023: 5-024) and intracranial measuring probe (OPS-2023: 5-029), and others. A prominent black dashed line signifies the cumulative total of these interventions, providing an encompassing view of the surgical treatment trends over the years. *CSF* cerebrospinal fluid.

Overall, surgical interventions for normal pressure hydrocephalus had an 8.4% increase, from 1346 in 2005 to 1459 in 2022 (Fig. 3). The peak was in 2017, with 1943 procedures recorded. A variety of interventions were recorded, including CSF shunts, Intracranial measuring probes, and other CSF diversion procedures.

### CSF shunts inserted

**Fig. 4:**
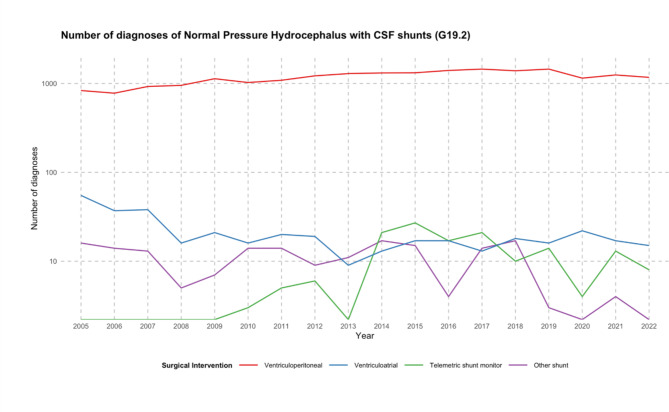
The line chart depicts the progression of normal pressure hydrocephalus diagnoses (categorized by ICD-10 code G91.2) with different types of CSF shunts between 2005 and 2022. The options are classified into seven shunt categories: Ventriculoperitoneal (OPS-2023: 5-023.1), ventriculoatrial (OPS-2023: 5-023.0), telemetric (OPS-2023: 5-023.3), and other shunts (OPS-2023: 5-023.x).

From 2005 to 2022, overall CSF shunt insertion saw a rise of 32.1%. By the end of this period, in 2022, there were a total of 1,194 such procedures conducted annually (as depicted in Fig. 3). Shunt removal and revision saw a decrease of 18.5% in the same period, from 178 in 2005 to 145 in 2022. The ratio of CSF shunt: Revision and removal of shunts increased by 59.3% over the 18 years. The most common shunt choice was a ventriculoperitoneal approach, accounting for 96.8% of all CSF shunts implemented in the study period (Table 1). From 2005 to 2022, overall VP shunt insertion saw a rise of 40.6%. By the end of this period, in 2022, there were a total of 1171 such procedures (as depicted in Fig. 4). Conversely, diagnoses related to VA shunts decreased by 72.7% at the same time.

### Other surgical operations

**Fig. 5:**
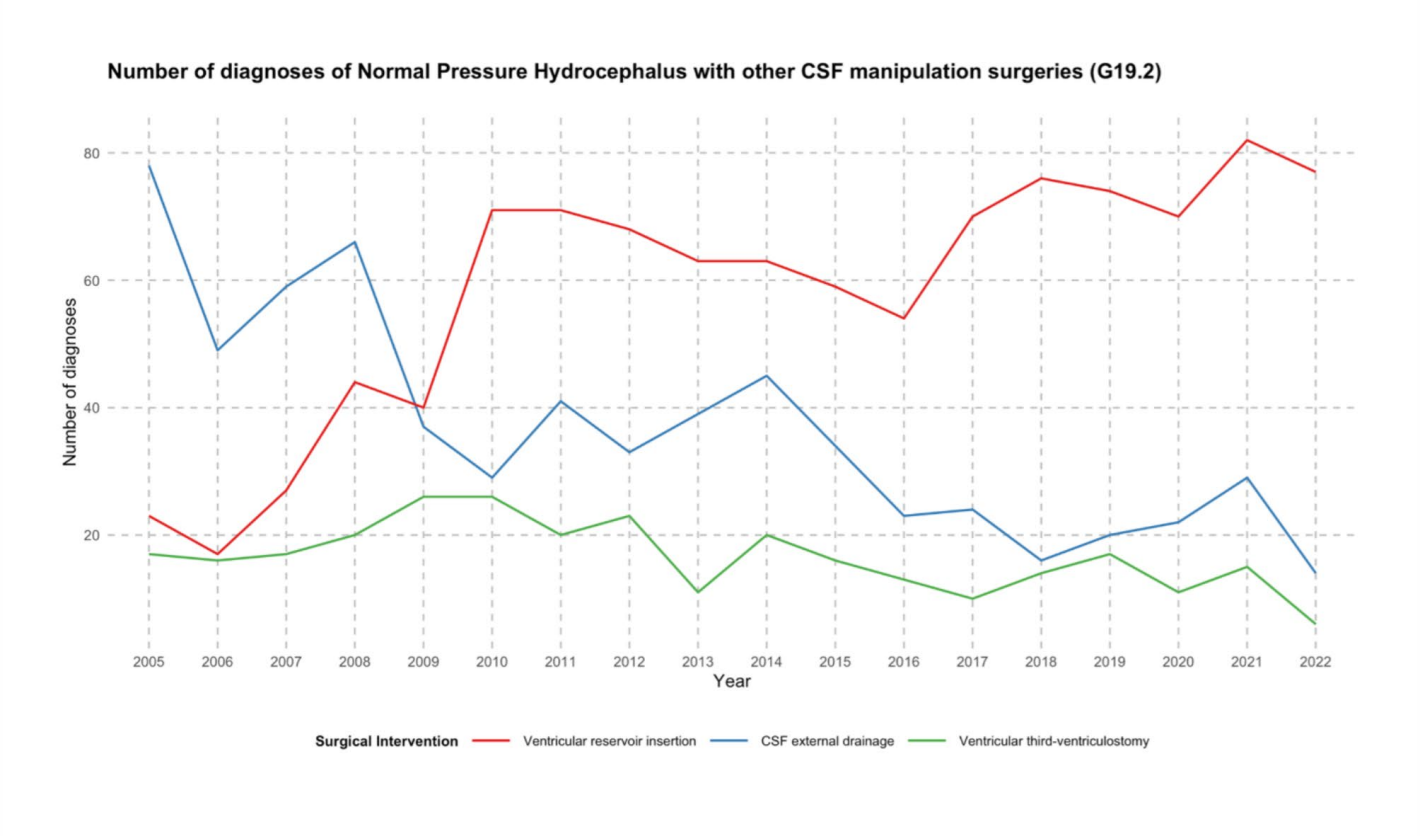
The depicted line chart illustrates the number of other surgical operations for Normal pressure Hydrocephalus (categorized by ICD-10 code: G91.2) with different types of operations between 2005 and 2022. They are classified into three categories: Ventricular reservoir insertion (OPS-2023: 5-022.1), CSF external drainage (OPS-2023: 5-022.0) and endoscopic third-ventriculostomy (OPS-2023: 5-02).

In addition to shunt insertion, other surgeries included: CSF external drainage, ventricular reservoir insertions and endoscopic third-ventriculostomy. From 2005 to 2022, there was a decrease in these procedures by 17.7%, from 124 to 102 cases (Fig. 5). Overall, Ventricular reservoir insertion was the most common out of the three accounting for 1049 procedures, followed by CSF external drainage with 658 and endoscopic third-ventriculostomy with 298 (Table 2).

## Discussion

Normal pressure hydrocephalus represents a significant yet under-recognized neurological disorder, particularly among the elderly, and prior to this study, the exact incidence within Germany remained undefined. The condition’s reversible nature contrasts starkly with its potential to lead to severe morbidity if not promptly and effectively managed^11^. This study is the first to harness data from the German Federal Database Statistics (Destatis) to evaluate the recent epidemiology of NPH in Germany, revealing a 48% increase in recorded cases between 2005 and 2022, from 5.5 per 100,000 population in 2005 to 8.0 per 100,000 population in 2022, with the most significant rise noted in the most vulnerable, namely the age group 80–89 years.

Our analysis shows that the incidence of NPH diagnosis in Germany has increased significantly over the last 18 years. Despite accounting for the ageing and growing population, we found that the number of cases rose by almost 50%. This is a much higher incidence rate than previously estimated in Germany, that ranged from 1.09-5/100,000/year. In comparison, the overall incidence of inpatient admission for iNPH was estimated to be 2.86/100,000 in the US^12^. The low incidence estimated rates reported in other studies may have been due to their study type, which is on selected subsample populations or are hospital-based^13^. Our nationwide registry study provides a more accurate depiction of iNPH diagnosis trends in Germany over the long term.

It is interesting to note that our study has shown only an increase in NPH diagnosis was observed in the three oldest age groups (70–79, 80–89 and 90+), accounting for the overall increase during the study period^22^. The most dramatic increase was specifically observed among individuals aged 80–90, likely reflecting shifts in clinical awareness and diagnostic practices rather than any biological change. Elderly patients are now more frequently assessed for conditions like NPH—a level of attention they may not have received two decades ago. This trend highlights important developments in geriatric neurosurgery: an increasing focus on identifying and managing treatable conditions in older adults to improve their quality of life and functional independence. Conversely, groups below the age of 70 have had decreased incidences. Moreover, these age groups have also shown an increase in incidence that exceeded the growth attributable solely to expected demographic changes in their respective population size. These shifts could stem from improvements in diagnostic modalities, alterations in healthcare availability, and fluctuations in the prevalence of comorbidities^14^. Our results imply that factors beyond mere demographic shifts contribute to the increasing incidence of this condition, particularly among the elderly demographic, therefore highlighting a pivotal storyline in the epidemiology of normal pressure hydrocephalus.

The observed decline in NPH diagnoses from 2019 to 2021 warrants attention, as it potentially reflects the extensive impact of the COVID-19 pandemic on healthcare systems globally^15^. The pandemic has had a multifaceted effect on medical care, deterring individuals from seeking hospital treatment due to infection fears and overwhelming healthcare facilities, leading to a reduction in routine medical services^16^. Consequently, this period likely saw a significant number of undiagnosed or deferred NPH cases due to these capacity constraints^17^. Given that NPH requires timely diagnosis and management to prevent irreversible neurological deterioration^18^, there is a concern for a substantial latent backlog of patients. These individuals remain untreated and may present a substantial ‘grey number’ within the German healthcare system, poised to place further strain^19^ on already taxed neurological and neurosurgical services as restrictions ease. This backlog represents not only a looming clinical challenge but also emphasizes the necessity for robust healthcare strategies to address the anticipated surge in demand for NPH-related healthcare services in the coming years.

Over the duration of our study, a surge was observed in the number of patients undergoing surgical intervention as part of their normal pressure hydrocephalus management. This surge in surgical interventions reflects the burgeoning adoption of evidence-based practices, bolstered by several published guidelines, enhanced patient selection criteria and better awareness of the condition among referring physicians^20^. Mechanical complications associated with CSF shunt devices, exacerbated by patient-related factors, continue to be significant determinants for shunt revision surgeries^21^. Predominantly, the majority of revisions are attributed to underdrainage (57–66%) and shunt infections (12–17%)^22^. This overarching decline in revision rates seen in this study highlights the contemporary advancements in both surgical methodologies and shunt device technologies in Germany, and possibly improved patient selection^23^. Furthermore, the introduction of programmable valves and devices for gravitational control and refined antibiotic administration protocols, have markedly reduced the incidence of complications associated with inappropriate over-drainage (a risk factor for subdural hematomas) and CNS infection thereby reducing the need to remove or manipulate primary shunts^24^.

While our study leverages extensive data from the Federal Statistical Office, it’s essential to recognize potential biases due to varying reporting standards across institutions. This is especially important consider the variations and difficulties in diagnosing NPH, which is frequently mistaken for other neurodegenerative disorders^25^. The introduction of the DRG system in Germany in 2004 could have influenced the early years of our dataset, reflecting an adaptation in hospitals’ coding and reporting^26^. Such variations might lead to potential underrepresentation or misclassification of cases at certain centers. Although our data captures data from hospitals, other confounding factors, like regional healthcare disparities or demographic shifts, could also influence our results. ICD-10 and OCD codes are vital for standardizing recording medical procedures across different centers in a region and facilitate streamline retrieval identification for in retrospective analysis. It is important to acknowledge their inherent limitations including misclassification and missing/unclear data reported under ambiguous and non-specific codes. To mitigate this, we diligently incorporated these ambiguous codes in our data and analysis to offer for a comprehensive portrayal of the state of normal pressure hydrocephalus within Germany. Furthermore, the absence of follow-up data in the database means that analysis was not possible of the shunt efficacy and surgical complications incidences.

## Conclusion

Our detailed analysis leveraging data from the Federal Statistical Office from 2005 to 2022 has identified a pronounced increase in the incidence of normal pressure hydrocephalus (NPH) in Germany, with an almost 50% escalation to 8.0 cases per 100,000 individuals. This rise transcends demographic trends, signifying a true increase in NPH occurrences, particularly among the elderly—a group that now represents the bulk of this condition’s burden, while the younger population shows a reduction in incidence. This study also reports an increase in surgical management of NPH, notably using ventriculoperitoneal shunt surgery, with a notable decrease in shunt revisions, reflecting advancements in surgical techniques and patient management protocols. These findings point to the criticality of refined diagnostic capabilities and management strategies for NPH, as well as empirical evidence on the diagnosis and management of NPH. They also compel further examination into the causative factors driving the increase in NPH incidence and a comprehensive evaluation of surgical outcomes for managing this condition. The current landscape necessitates that German healthcare policymakers and the scientific community accord high priority to NPH, focusing on optimizing care pathways, addressing the backlog of undiagnosed cases post-pandemic, and enhancing research efforts to mitigate the rising epidemiological and clinical impact of NPH on the healthcare system.

## Data Availability

All relevant data supporting the findings of this study can be accessed from our dedicated GitHub repository. For ensuring transparency, replicability, and rigorousness of the research, this repository encompasses both the raw and processed data integral to our study. Access the dataset via the following link: Access the complete R code used in this study via the following link: https://github.com/ahmedsalih12/NPHepidemiology. We strongly encourage fellow researchers and individuals with vested interests to employ these resources in their respective research endeavours and subsequent analyses.

## Abbreviations

ANOVA: Analysis of variance
CNS: Central nervous system
CSF: Cerebrospinal fluid
Destatis: Federal Statistical Office of Germany
DRG: Diagnosis-related group
ICD: International classification of diseases
iNPH: Idiopathic normal pressure hydrocephalus
NPH: Normal pressure hydrocephalus
OPS: Operation and procedure code
VA: Ventriculoatrial
VP: Ventriculoperitoneal
